# Divergent roles of estrogen receptor subtypes in regulating estrogen-modulated colonic ion transports and epithelial repair

**DOI:** 10.1016/j.jbc.2023.105068

**Published:** 2023-07-17

**Authors:** Hanxing Wan, Junhui Li, Xiongying Chen, Zachary M. Sellers, Hui Dong

**Affiliations:** 1 Department of Pharmacology, School of Pharmacy, Qingdao University Medical College, Qingdao, China; 2 Department of Gastroenterology, Xinqiao Hospital, Army Medical University, Chongqing, China; 3 Pediatric Gastroenterology Hepatology & Nutrition, Stanford University School of Medicine, Palo Alto, California, USA

**Keywords:** ERα subtype, ERβ subtype, intestinal ion transport, colonic epithelial restitution

## Abstract

Although it was described previously for estrogen (E_2_) regulation of intestinal epithelial Cl^−^ and HCO_3_^−^ secretion in sex difference, almost nothing is known about the roles of estrogen receptor (ER) subtypes in regulating E_2_-modulated epithelial ion transports and epithelial restitution. Here, we aimed to investigate ERα and ERβ subtypes in the regulation of E_2_-modulated colonic epithelial HCO_3_^−^ and Cl^−^ secretion and epithelial restitution. Through physiological and biochemical studies, in combination of genetic knockdown, we showed that ERα attenuated female colonic Cl^−^ secretion but promoted Ca^2+^-dependent HCO_3_^−^ secretion *via* store-operated calcium entry (SOCE) mechanism in mice. However, ERβ attenuated HCO_3_^−^ secretion by inhibiting Ca^2+^*via* the SOCE and inhibiting cAMP *via* protein kinases. Moreover, ERα but not ERβ promoted epithelial cell restitution *via* SOCE/Ca^2+^ signaling. ERα also enhanced cyclin D1, proliferating cell nuclear antigen, and β-catenin expression in normal human colonic epithelial cells. All ERα-mediated biological effects could be attenuated by its selective antagonist and genetic knockdown. Finally, both ERα and ERβ were expressed in human colonic epithelial cells and mouse colonic tissues. We therefore conclude that E_2_ modulates complex colonic epithelial HCO_3_^−^ and Cl^−^ secretion *via* ER subtype-dependent mechanisms and that ERα is specifically responsible for colonic epithelial regeneration. This study provides novel insights into the molecular mechanisms of how ERα and ERβ subtypes orchestrate functional homeostasis of normal colonic epithelial cells.

Epithelial ion transports are pivotal physiological process in several human organs, such as gastroenterological (GI) tract, respiratory tract, reproductive tract, and skin. Intestinal epithelium either absorbs electrolytes or secretes anions (such as HCO_3_^−^ and Cl^−^), providing the driving force for fluid transport to maintain fluid homeostasis in human body (1, 2). Intestinal epithelial anion secretion is under control of endogenous neurohumoral factors, such as acetylcholine, prostaglandin E_2_, 5-HT, and nitric oxide. These neurohumoral factors trigger intracellular Ca^2+^, cAMP, and cGMP signaling to mediate intestinal epithelial anion secretion *via* plasma membrane ion channels and transporters, such as cystic fibrosis transmembrane conductance regulator and Cl^−^/HCO_3_^−^ exchanger (*i.e.*, DRA) (3, 4), etc.

The duodenal mucosa not only senses luminal nutrients but also regulates ion transports (particularly HCO_3_^−^ and Cl^−^ secretion), which in turn is important for nutrient absorption and mucosal protection (5, 6). It was reported that patients with duodenal ulcer had significantly diminished proximal duodenal HCO_3_^−^ secretion compared with healthy volunteers (7, 8), suggesting not only that normal duodenal HCO_3_^−^ secretion is pivotal to mucosal protection but that diminished duodenal HCO_3_^−^ secretion contributes to duodenal ulcer. We previously revealed a sex difference in duodenal HCO_3_^−^ secretion in mice and found the expression and function of estrogen receptors (ER) in murine duodenal epithelium (9). We further demonstrated that estrogen (E_2_) may protect human duodenum against the acid-induced injury by mediating duodenal HCO_3_^−^ secretion likely *via* ER activation, which explains the lower incidence of duodenal ulcer in women than age-matched men (10). However, it is largely unknown about the involvement of different ER subtypes (ERα and ERβ) in the process of intestinal epithelial HCO_3_^−^ secretion, let alone the underlying mechanisms of ERα- and ERβ-mediated HCO_3_^−^ secretion.

Colonic epithelial anion secretion is a well-established physiological process closely linked to overall fluid and electrolyte movement in the colon (11, 12, 13). It is vital to maintain normal colonic HCO_3_^−^ secretion, which loss (such as in diarrhea) may cause not only imbalance of pH values and electrolytes in whole body but also local disruption of colonic environments, such as epithelial barrier and microbiome (11, 12, 13). Therefore, the studies on colonic HCO_3_^−^ secretion may offer an opportunity for improving human GI health. Unfortunately, it has not been explored if E_2_ regulates colonic HCO_3_^−^ secretion *via* ER activation so far; and if so, what ER subtypes and mechanisms are involved.

While E_2_ promotes duodenal HCO_3_^−^ secretion (9, 10), it inhibits colonic Cl^−^ secretion in sex difference (14, 15, 16). However, the underlying mechanisms are unclear, and it is even unknown if E_2_ inhibition of Cl^−^ secretion is *via* ER or not. Moreover, integrity and homeostasis of intestinal mucosa are crucial for GI function, which depends upon the balance between mucosal injury and healing (17). Although epithelial cell restitution plays an important role in healing process (17), little is known about the involvement of ER subtypes in colonic mucosal healing. Therefore, in the present study, we hypothesized that ER subtypes may play different roles in the regulation of E_2_-modulated colonic HCO_3_^−^ and Cl^−^ secretion, and we examined the underlying mechanisms of ER subtypes in E_2_-mediated colonic epithelial HCO_3_^−^ secretion and epithelial repair.

## Results

### ERα stimulation of HCO_3_^−^ secretion from the male duodenum and distal colon

**Figure 1:**
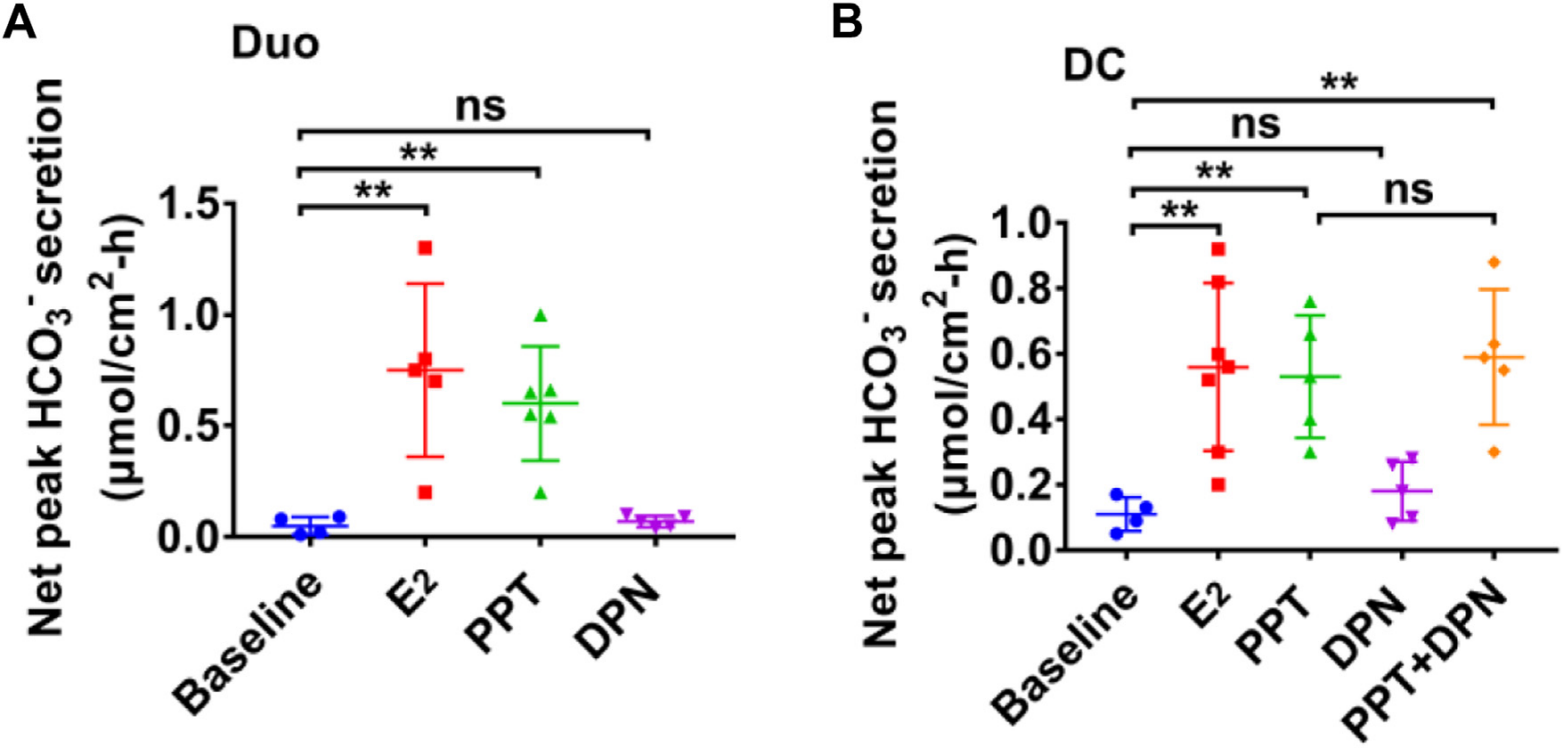
**Activation of ERα but not ERβ stimulates epithelial HCO**_**3**_^**−**^**secretion in both duodenum and distal colon.***A*, effects of estradiol-17β (E_2_, 100 nM, n = 6), propyl pyrazole triol (PPT, 10 nM, n = 6), and diarylpropionitrile (DPN, 10 nM, n = 5) on duodenal net peak HCO_3_^−^ secretion. *B*, effects of E_2_ (100 nM, n = 7), PPT (10 nM, n = 5), DPN (10 nM, n = 5) and PPT plus DPN (n = 5) on distal colonic net peak HCO_3_^−^ secretion. E_2_, PPT, and DPN were added to both sides, but CCh and forskolin were add to serosal side. ∗∗*p* < 0.01. ns, no significant differences. Data are presented as mean ± SD. The statistical significance of differences in the means of experimental groups was determined using Student's *t* test or one-way ANOVA followed by post hoc test for multiple pairwise comparisons. CCh, carbachol; ER, estrogen receptor.

Since the lack of information on ER subtypes in intestinal HCO_3_^−^ secretion, we performed Ussing chamber experiments with pH-stat to examine the roles of ER subtypes in HCO_3_^−^ secretion from the duodenum and distal colon in male mice. First, we found that both estradiol-17β (E_2_, 100 nM) and ERα selective activator propyl pyrazole triol (PPT, 10 nM) stimulated rapid duodenal HCO_3_^−^ secretion; however, ERβ selective activator diarylpropionitrile (DPN, 10 nM) did not (Fig. 1*A*). Second, we tested if there is any regional heterogeneity for the ER subtype-mediated HCO_3_^−^ secretion in between the duodenum and distal colon. Like in the duodenum, both E_2_ and PPT stimulated colonic HCO_3_^−^ secretion but DPN alone did not (Fig. 1*B*). Moreover, compared to PPT alone, PPT plus DPN did not further stimulate additional colonic HCO_3_^−^ secretion (Fig. 1*B*). Thus, ERα activation stimulated HCO_3_^−^ secretion from the duodenum and distal colon, which has no regional heterogeneity in GI tract. In the subsequent experiments, distal colonic HCO_3_^−^ secretion was focused because duodenal HCO_3_^−^ secretion has been extensively studied (10, 18, 19).

### Differing effects of ERα and ERβ receptors on Ca^2+^-mediated and cAMP-mediated colonic HCO_3_^−^ secretion

**Figure 2:**
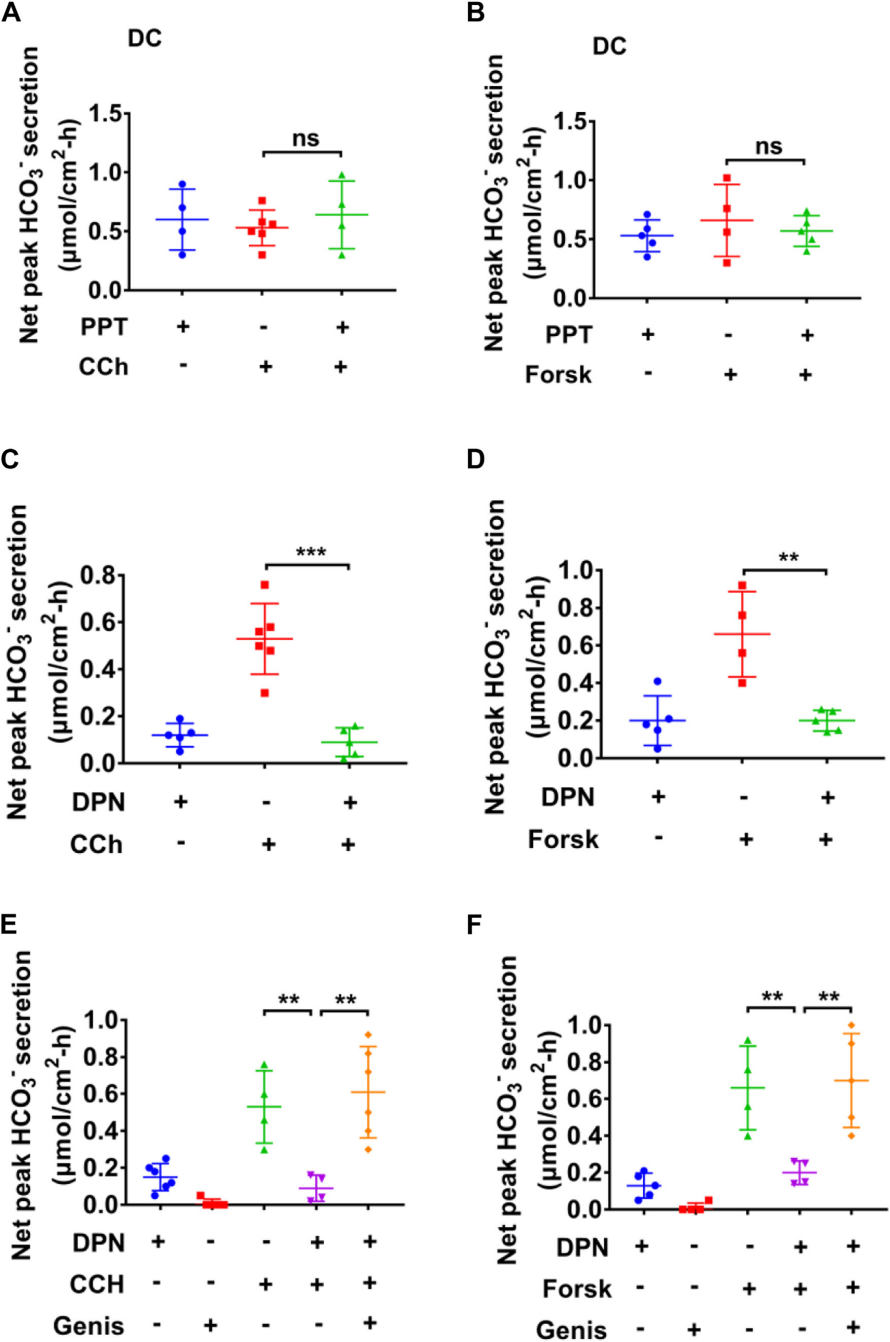
**Activation of ERβ but not ERα inhibits carbachol- and forskolin-induced male colonic HCO**_**3**_^**−**^**secretion *via* inhibition of CFTR.***A* and *B*, effect of PPT (10 nM) on carbachol (CCh, 100 μM)- and forskolin (Forsk, 20 μM)-induced colonic HCO_3_^−^ secretion. *C* and *D*, effect of DPN (10 nM) on CCh- and Forsk-induced colonic HCO_3_^−^ secretion. *E* and *F*, genistein (Genis, 20 μM) reversed DPN (10 nM)-inhibited CCh- and Forsk-induced colonic HCO_3_^−^ secretion. PPT, DPN, and genistein were add to both sides, but CCh and forskolin were added to serosal side. ∗∗*p* < 0.01 and ∗∗∗*p* < 0.001. n = 5 to 6 tissues for each group. ns, no significant differences. Data are presented as mean ± SD. The statistical significance of differences in the means of experimental groups was determined using Student's *t* test. CFTR, cystic fibrosis transmembrane conductance regulator; DPN, diarylpropionitrile; ER, estrogen receptor; PPT, propyl pyrazole triol.

It is well-known that Ca^2+^ and cAMP signaling pathways play crucial roles in regulating intestinal anion secretion (11, 12, 13, 18, 19, 20, 21). Since carbachol (CCh, a cholinergic agonist) and forskolin (an adenylcyclase activator) stimulate colonic anion secretion by triggering intracellular Ca^2+^ and cAMP signaling respectively (15), we examined if ER signaling may impact CCh (Ca^2+^ signaling)- or forskolin (cAMP signaling)-stimulated HCO_3_^−^ secretion. As shown in Fig. 2, *A* and *B*, the selective ERα activator PPT (10 nM) pretreatment did not affect CCh (100 μM)- or forskolin (20 μM)-induced colonic HCO_3_^−^ secretion, indicating that ERα-stimulated HCO_3_^−^ secretion did not utilize the same signaling pathways as CCh or forskolin. In contrast, although the selective ERβ activator DPN (10 nM) did not alter basal colonic HCO_3_^−^ secretion, it almost abolished both CCh (100 μM)- and forskolin (20 μM)-induced colonic HCO_3_^−^ secretion (Fig. 2, *C* and *D*), suggesting that ERβ activation may alter Ca^2+^- and cAMP-mediated colonic HCO_3_^-^ secretion.

### ERβ inhibits male colonic HCO_3_^−^ secretion *via* protein kinase pathways

After demonstrating ERβ inhibition of CCh- and forskolin-induced colonic HCO_3_^−^ secretion, we further elucidated the underlying mechanisms. Since protein kinase, such as tyrosine kinase (TK), protein kinase C (PKC), and phosphoinositide 3-kinase (PI3K) pathways play critical roles in intestinal HCO_3_^−^ secretion (6, 22, 23, 24, 25), we next studied the intracellular signaling mechanisms behind ERβ inhibition of Ca^2+^- and cAMP-stimulated HCO_3_^−^ secretion. First, as shown in Figure 2, *E* and *F*, genistein (20 μM), a commonly used TK inhibitor, inhibited basal HCO_3_^−^ secretion, suggesting its role in basal HCO_3_^−^ secretion, consistently with the report by Osamu Furukawa *et al* (25). Furthermore, genistein reversed DPN inhibition of CCh- and forskolin-induced HCO_3_^-^ secretion (Fig. 2, *E* and *F*), suggesting that ERβ activation inhibits Ca^2+^- and cAMP-mediated colonic HCO_3_^−^ secretion likely *via* TK pathway. Although genistein may also activate ER (26), at 20 μM, it inhibited rather than stimulated basal HCO_3_^-^ secretion, excluding the possibility that genistein at this concentration stimulates HCO_3_^−^ secretion *via* ER activation.

**Figure 3:**
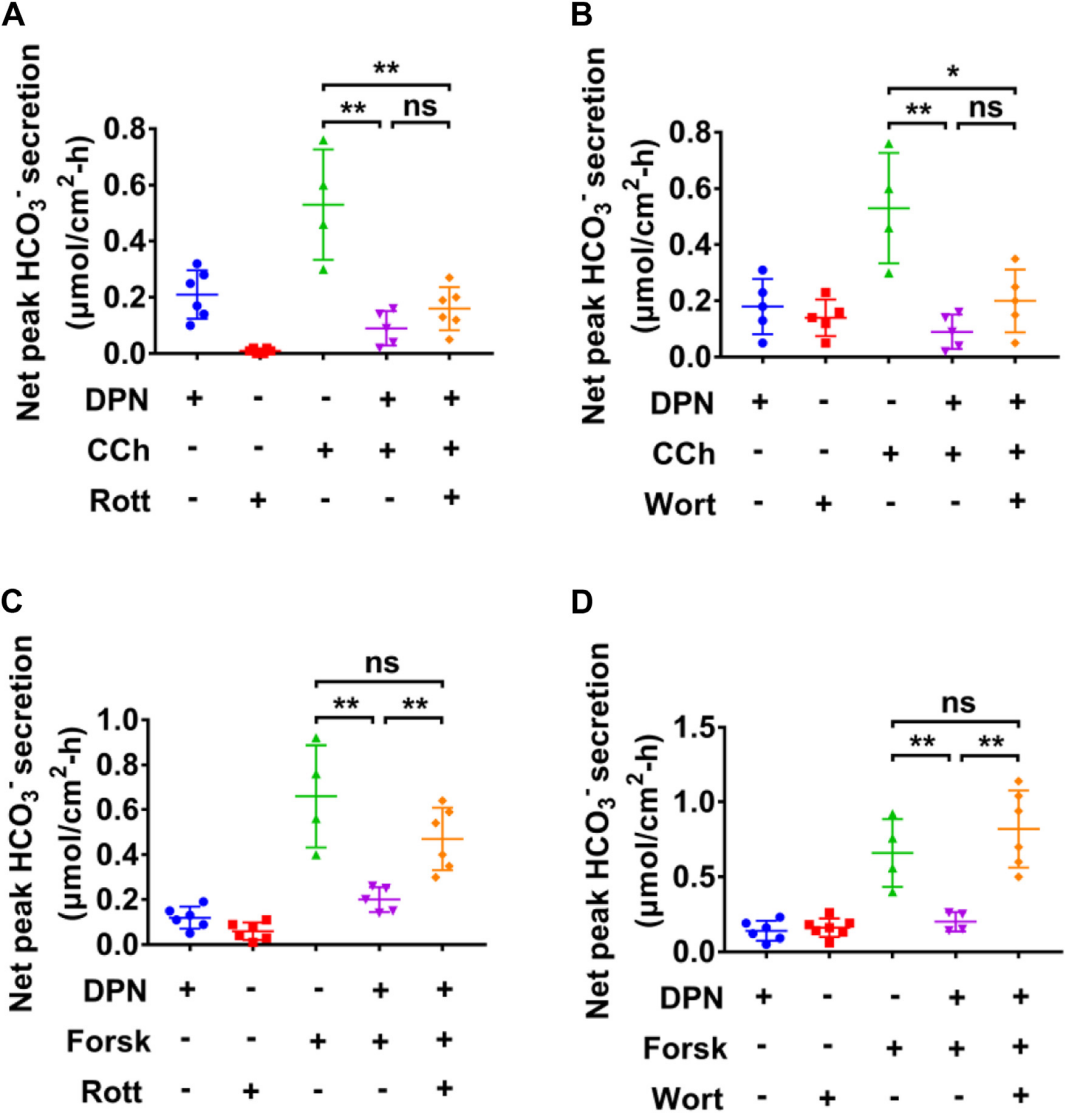
**ERβ activation inhibits carbachol- and forskolin-stimulated male colonic HCO**_**3**_^**−**^**secretion *via* different signaling pathways.***A*, rottlerin (Rott, 10 μM) did not affect DPN (10 nM)-inhibited CCh (100 μM)-stimulated HCO_3_^−^ secretion. DPN (n = 6), rottlerin (n = 6), CCh (n = 4), DPN + CCh (n = 5), and rottlerin + DPN + CCh (n = 6). *B*, Wortmannin (Wort, 100 nM) did not affect DPN (10 nM)-inhibited CCh (100 μM)-stimulated HCO_3_^−^ secretion. DPN (n = 5), wortmannin (n = 5), CCh (n = 4), DPN + CCh (n = 5), and wortmannin + DPN + CCh (n = 5). *C*, rottlerin (10 μM) reversed DPN (10 nM)-inhibited forskolin (Forsk, 20 μM)-stimulated HCO_3_^−^ secretion. DPN (n = 6), rottlerin (n = 6), forskolin (n = 4), DPN + forskolin (n = 5), and rottlerin + DPN + forskolin (n = 6). *D*, wortmannin (100 nM) reversed DPN (10 nM)-inhibited forskolin (20 μM)-stimulated HCO_3_^−^ secretion. DPN (n = 6), wortmannin (n = 7), forskolin (n = 4), DPN + forskolin (n = 4), and wortmannin + DPN + forskolin (n = 6). PPT, DPN, rottlerin, and wortmannin were added to both sides, but CCh and forskolin were added to serosal side. ∗*p* < 0.05 and ∗∗*p* < 0.01. ns, no significant differences. Data are presented as mean ± SD. The statistical significance of differences in the means of experimental groups was determined using Student's *t* test or one-way ANOVA followed by post hoc test for multiple pairwise comparisons. CCh, carbachol; DPN, diarylpropionitrile; ER, estrogen receptor; PPT, propyl pyrazole triol.

Second, rottlerin (10 μM), a selective PKC inhibitor (27), attenuated basal HCO_3_^−^ secretion (Fig. 3, *A* and *C*), suggesting its role in regulating basal HCO_3_^−^ secretion, which is consistent with the report by Odes HS *et al* (26). However, a selective PI3K inhibitor wortmannin (100 nM) (24) did not alter basal HCO_3_^−^ secretion (Fig. 3, *B* and *D*). Neither rottlerin nor wortmannin affected DPN inhibition of CCh-induced male colonic HCO_3_^−^ secretion (Fig. 3, *A* and *B*). In contrast to CCh-induced colonic HCO_3_^−^ secretion, both rottlerin and wortmannin reversed DPN inhibition of forskolin-induced colonic HCO_3_^−^ secretion (Fig. 3, *C* and *D*). These data suggest that ERβ activation inhibits cAMP-mediated colonic HCO_3_^−^ secretion *via* PKC and PI3K pathways, which are not involved in ERβ inhibition of Ca^2+^-mediated HCO_3_^−^ secretion.

### ERα specifically inhibits CCh- and forskolin-induced female colonic short-circuit current

**Figure 4:**
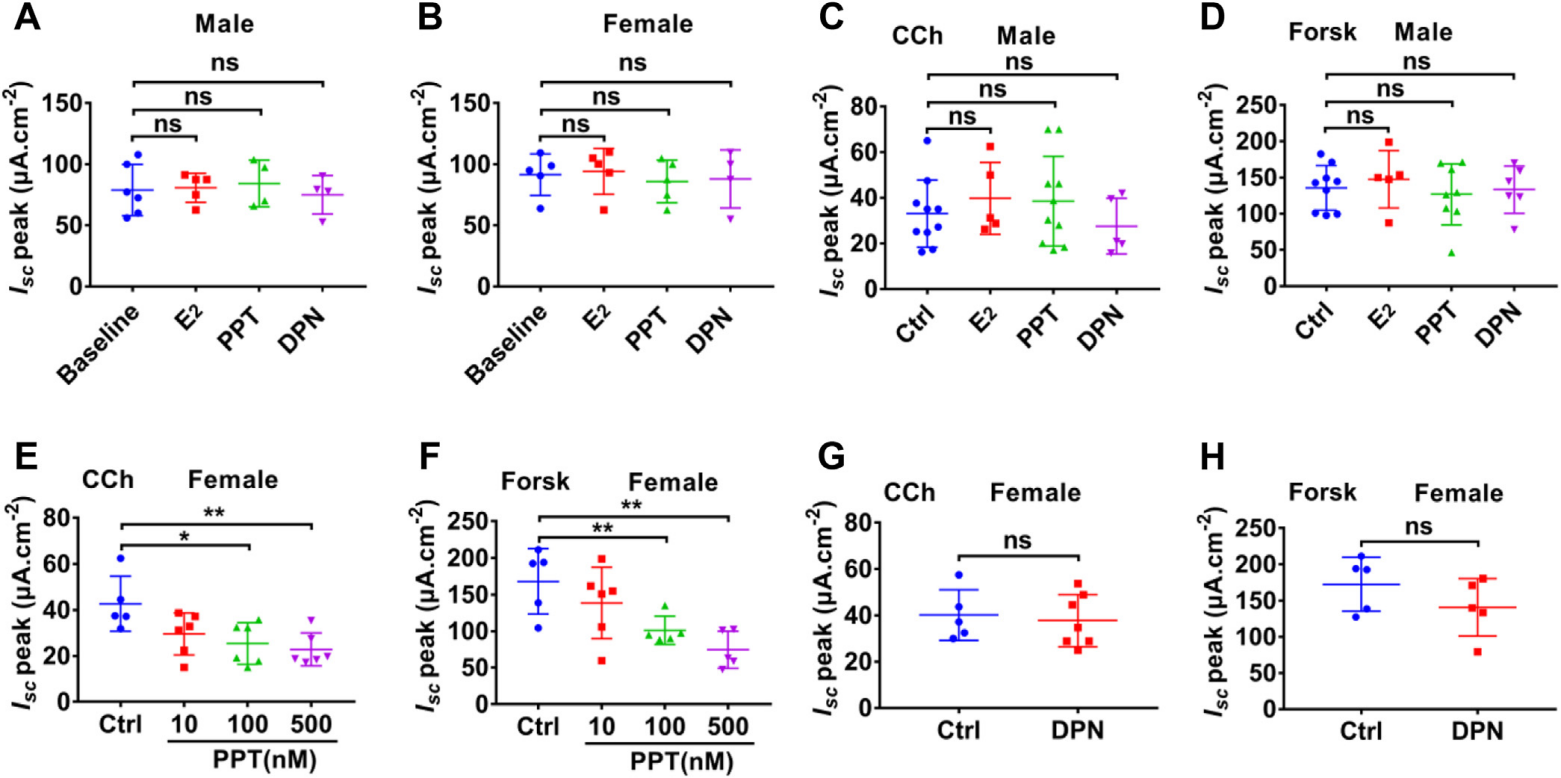
**ERα activation specifically inhibited carbachol- or forskolin-induced female colonic *I***_***sc***_. *A*, E_2_ (1 μM), PPT (500 nM), and DPN (500 nM) did not affect basal colonic short-circuit current (*I*_*sc*_) in male mice. Baseline (n = 6), E_2_ (n = 5), PPT (n = 4), and DPN (n = 4). *B*, E_2_ (1 μM), PPT (500 nM), and DPN (500 nM) did not affect basal colonic short-circuit current (*I*_*sc*_) in female mice. Baseline (n = 5), E_2_ (n = 5), PPT (n = 5), and DPN (n = 4). *C*, E_2_ (1 μM), PPT (500 nM), and DPN (500 nM) did not affect carbachol (CCh, 50 μM)-induced male colonic *I*_*sc*_. Control (Ctrl, n = 10), E_2_ (n = 5), PPT (n = 10), and DPN (n = 5). *D*, E_2_, PPT, and DPN did not affect forskolin (Forsk, 5 μM)-induced male colonic *I*_*sc*_, Control (Ctrl, n = 9), E_2_ (n = 5), PPT (n = 8), and DPN (n = 6). *E*, PPT inhibited CCh (50 μM)-induced *I*_*sc*_ of female mouse colon, Ctrl (n = 5), PPT (10 nM, n = 6), PPT (100 nM, n = 6), and PPT (500 nM, n = 6). *F*, PPT inhibited forskolin (Forsk, 5 μM)-induced *I*_*sc*_ of female mouse colon, Ctrl (n = 5), PPT (10 nM, n = 6), PPT (100 nM, n = 5), and PPT (500 nM, n = 5). *G*, DPN (500 nM) did not affect CCh (50 μM)-induced *I*_*sc*_ of female mouse colon. Ctrl (n = 5) and DPN (n = 7). *H*, DPN (500 nM) did not affect forskolin (Forsk, 5 μM)-induced *I*_*sc*_ of female mouse colon. Ctrl (n = 5) and DPN (n = 5). Ctrl represents as the control with CCh or forskolin treatment only. PPT, DPN, and E_2_ were added to both sides. CCh and forskolin were added to serosal side. ∗*p* < 0.05 and ∗∗*p* < 0.01. ns, no significant differences. Data are presented as mean ± SD. The statistical significance of differences in the means of experimental groups was determined using Student's *t* test or one-way ANOVA followed by post hoc test for multiple pairwise comparisons. CCh, carbachol; DPN, diarylpropionitrile; ER, estrogen receptor; PPT, propyl pyrazole triol.

After demonstrating the roles and the mechanisms of ER subtypes in the regulation of male colonic HCO_3_^−^ secretion, we examined their roles in colonic Cl^−^ secretion that is poorly understood although it is known about E_2_ inhibition of Cl^−^ secretion in rat distal colonic epithelium with a gender-specific mechanism (14, 15, 28). None of E_2_ (1 μM), PPT (500 nM), and DPN (500 nM) at high concentrations affected basal colonic short-circuit current (*I*_*sc*_) in both sexes (Fig. 4, *A* and *B*). Moreover, none of them affected CCh- and forskolin-induced male colonic *I*_*sc*_ (Fig. 4, *C* and *D*), consistently with the previous reports that E_2_ did not alter colonic epithelial Cl^−^ secretion in male rats (15, 28).

We further examined the role of ER subtypes in female colonic Cl^−^ secretion. As shown in Figure 4, *E* and *F*, ERα selective activator PPT (100 nM) significantly inhibited CCh- and forskolin-induced *I*_*sc*_ of female mouse colon, which is consistent with the previous reports that E_2_ inhibited colonic epithelial Cl^−^ secretion through KCNQ1 channels in female rats (14, 15, 28). However, ERβ selective activator DPN at high concentration 500 nM did not affect CCh- and forskolin-induced *I*_*sc*_ of female mouse colon (Fig. 4, *G* and *H*). Taken together, ERα subtype is specifically responsible for the inhibition of female colonic *I*_*sc*_.

### ERα induces Ca^2+^ signaling *via* the store-operated Ca^2+^ entry, but ERβ inhibits the store-operated Ca^2+^ entry in human colonic epithelial cell line

**Figure 5:**
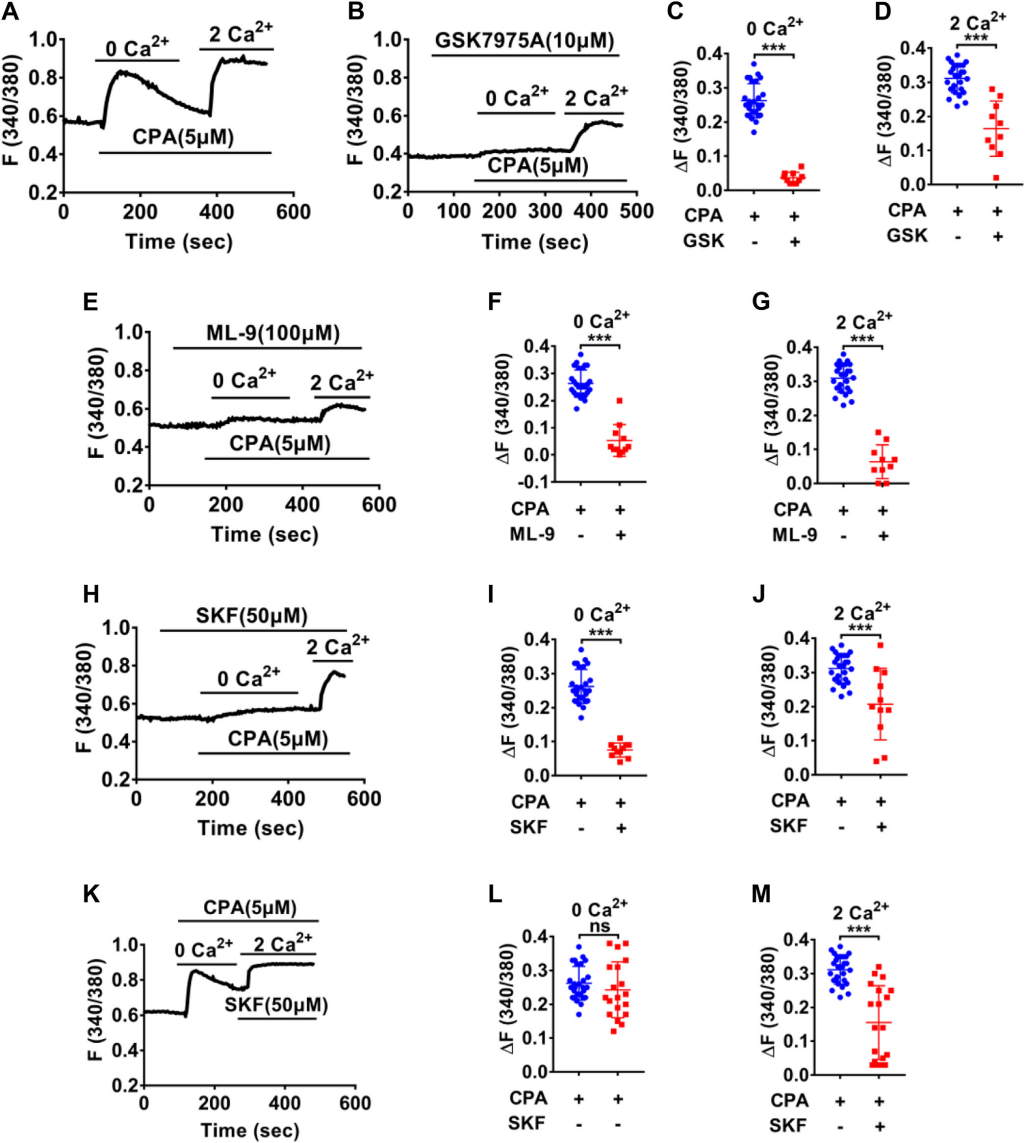
**Characterization of store-operated Ca**^**2+**^**entry (SOCE) function in HCoEpiC**. *A*, Summary tracings of [Ca^2+^]_cyt_ time course in response to cyclopiazonic acid (CPA) (5 μM, n = 27) in Ca^2+^-free solutions (0 Ca^2+^) and in Ca^2+^-containing solutions (2 Ca^2+^). *B, E* and *H*, summary tracings of [Ca^2+^]_cyt_ time course showing the inhibitory effect of GSK7975A (10 μM, n = 10), ML-9 (100 μM, n = 10), and SKF96365 (50 μM, n = 11) on CPA-induced Ca^2+^ release and Ca^2+^ influx. *C* and *D*, summary data showing the peaks of CPA-increased [Ca^2+^]_cyt_ signaling as described in (*A* and *B*). *F* and *G*, summary data showing the peaks of CPA-increased [Ca^2+^]_cyt_ signaling as described in (*A* and *E*). *I* and *J*, summary data showing the peaks of CPA-increased [Ca^2+^]_cyt_ signaling as described in (*A* and *H*). *K*, summary tracings of [Ca^2+^]_cyt_ time course showing the inhibitory effect of SKF96365 (50 μM, n = 19) on CPA-induced Ca^2+^ influx. *L* and *M*, summary data showing the peaks of CPA-increased [Ca^2+^]_cyt_ signaling as described in (*A* and *K*). ∗∗∗*p* < 0.001. ns, no significant differences. Data are presented as mean ± SD. The statistical significance of differences in the means of experimental groups was determined using Student's *t* test. [Ca2+]cyt, free cytoplasmic Ca2+; HCoEpiC, human colonic epithelial cell line.

It is well documented that Ca^2+^ signaling triggers intestinal HCO_3_^−^ secretion (9, 18, 20, 29), and E_2_ may induce HCO_3_^−^ secretion *via* Ca^2+^ signaling (9); however, little is known about ER-mediated colonic epithelial Ca^2+^ signaling. In other cell types, store-operated Ca^2+^ entry (SOCE) plays a pivotal role in controlling Ca^2+^ signaling. Initially, we applied single cell Ca^2+^ imaging to characterize the SOCE in human colonic epithelial cell line (HCoEpiC). Cyclopiazonic acid (CPA, 5 μM), an inhibitor of the sarcoendoplasmic reticulum Ca^2+^ ATPase and a commonly used SOCE activator, significantly induced free cytoplasmic Ca2+ [Ca^2+^]_cyt_ in Ca^2+^-free solutions and then a sustained [Ca^2+^]_cyt_ increase in Ca^2+^-containing solutions, suggesting that intracellular Ca^2+^ release causes extracellular Ca^2+^ influx in HCoEpiC (*i*.*e*, SOCE) (Fig. 5*A*). Pretreatment with three different SOCE blockers, GSK7975A (10 μM) (30), ML-9 (100 μM) (31), and SKF96365 (50 μM) (32), markedly eliminated the CPA-induced SOCE (Fig. 5, *B*–*J*), verifying SOCE function in HCoEpiC.

Although the initial Ca^2+^ transient produced by CPA in Ca^2+^-free solutions is presumably Ca^2+^ release from stores, it was reduced by pretreatment with SOCE blockers (Fig. 5, *C*, *F*, and *I*). We assume it is because the SOCE is important to refill the intracellular Ca^2+^ store, and Ca^2+^ release from stores would be reduced by pretreatment with SOCE blockers. To test this possibility, we added SOCE blockers after the store depletion but before extracellular Ca^2+^ influx. Indeed, SKF96365 (50 μM) markedly reduced CPA-induced Ca^2+^ influx (Fig. 5, *K*–*M*). These data verify not only SOCE function *per se* but also its important role in refilling intracellular Ca^2+^ store in HCoEpiC.

**Figure 6:**
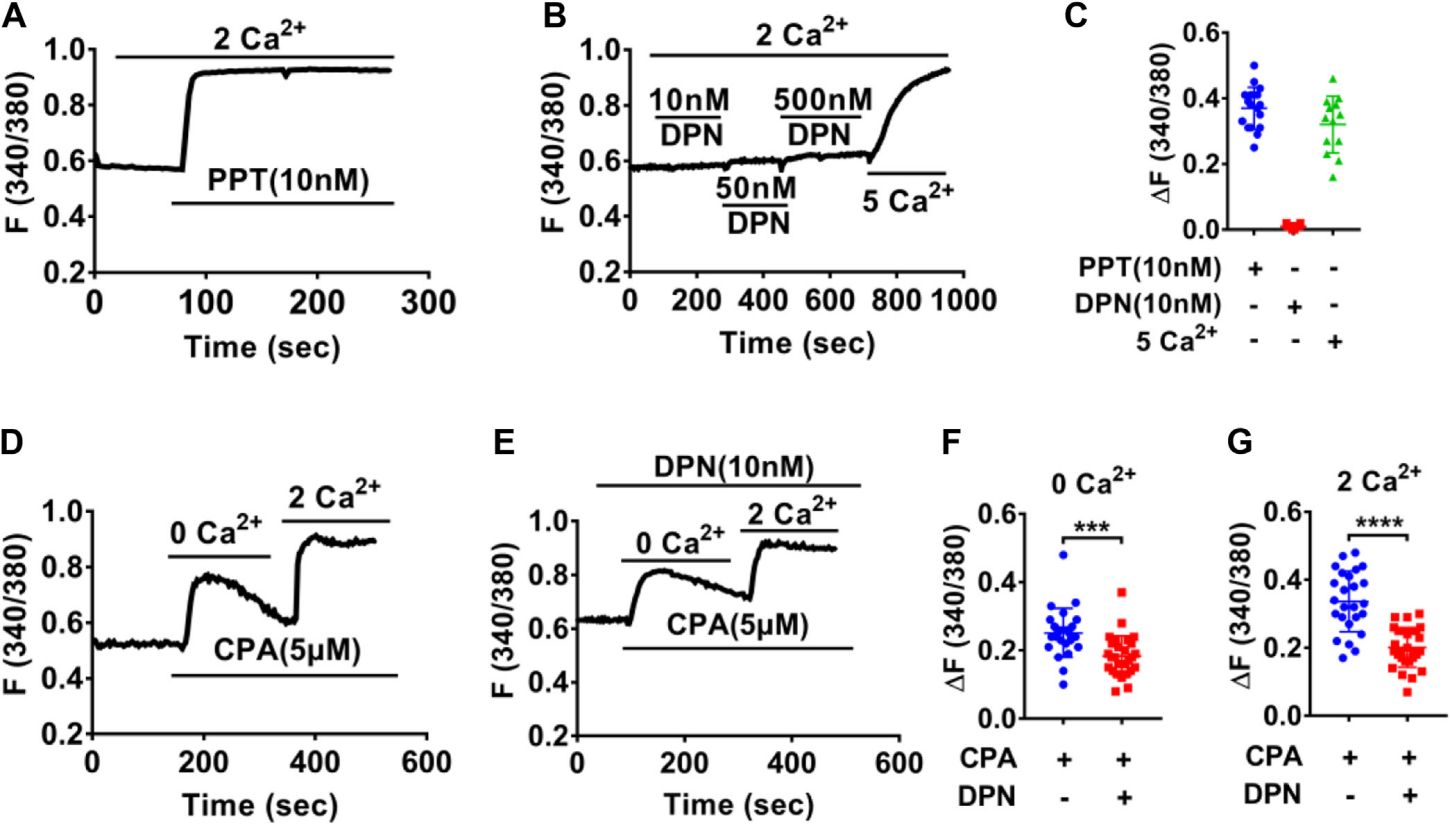
D**ifferential effects of ERα and ERβ activation on SOCE-mediated [Ca**^**2+**^**]**_**cyt**_**in HCoEpiC.***A*, summary tracings of [Ca^2+^]_cyt_ time course in response to PPT (10 nM, n = 17) in Ca^2+^-containing solutions. *B*, summary tracings of [Ca^2+^]_cyt_ time course in response to DPN (10–500 nM, n = 15) and 5 mM Ca^2+^ in Ca^2+^-containing solutions. *C*, summary data showing the peaks of PPT-, DPN- and 5 mM Ca^2+^-induced [Ca^2+^]_cyt_ signaling as described in (*A* and *B*). *D* and *E*, CPA (5 μM, n = 25)-induced Ca^2+^ release and Ca^2+^ influx, which was attenuated by DPN (10 nM, n = 28). *F* and *G*, summary data showing the peaks of CPA-increased [Ca^2+^]_cyt_ signaling with or without DPN as described in (*D* and *E*). ∗∗∗*p* < 0.001 and ∗∗∗∗*p* < 0.0001. ns, no significant differences. Data are presented as mean ± SD. The statistical significance of differences in the means of experimental groups was determined using Student's *t* test. [Ca^2+^]_cyt_, free cytoplasmic Ca^2+^; CPA, cyclopiazonic acid; DPN, diarylpropionitrile; ER, estrogen receptor; HCoEpiC, human colonic epithelial cell line; PPT, propyl pyrazole triol; SOCE, store-operated Ca^2+^ entry.

After characterizing the SOCE in HCoEpiC, we next examined whether ER stimulates colonic epithelial Ca^2+^ signaling. ERα selective activator PPT (10 nM) significantly stimulated [Ca^2+^]_cyt_ in Ca^2+^-containing solutions (Fig. 6, *A* and *C*), but ERβ selective activator DPN (10–500 nM) did not alter basal [Ca^2+^]_cyt_ (Fig. 6, *B* and *C*). However, DPN (10 nM) significantly inhibited CPA (5 μM)-induced SOCE (Fig. 6, *D*–*G*). Together, these data suggest that ERα triggers Ca^2+^ signaling, but ERβ inhibits Ca^2+^ signaling likely *via* the SOCE.

**Figure 7:**
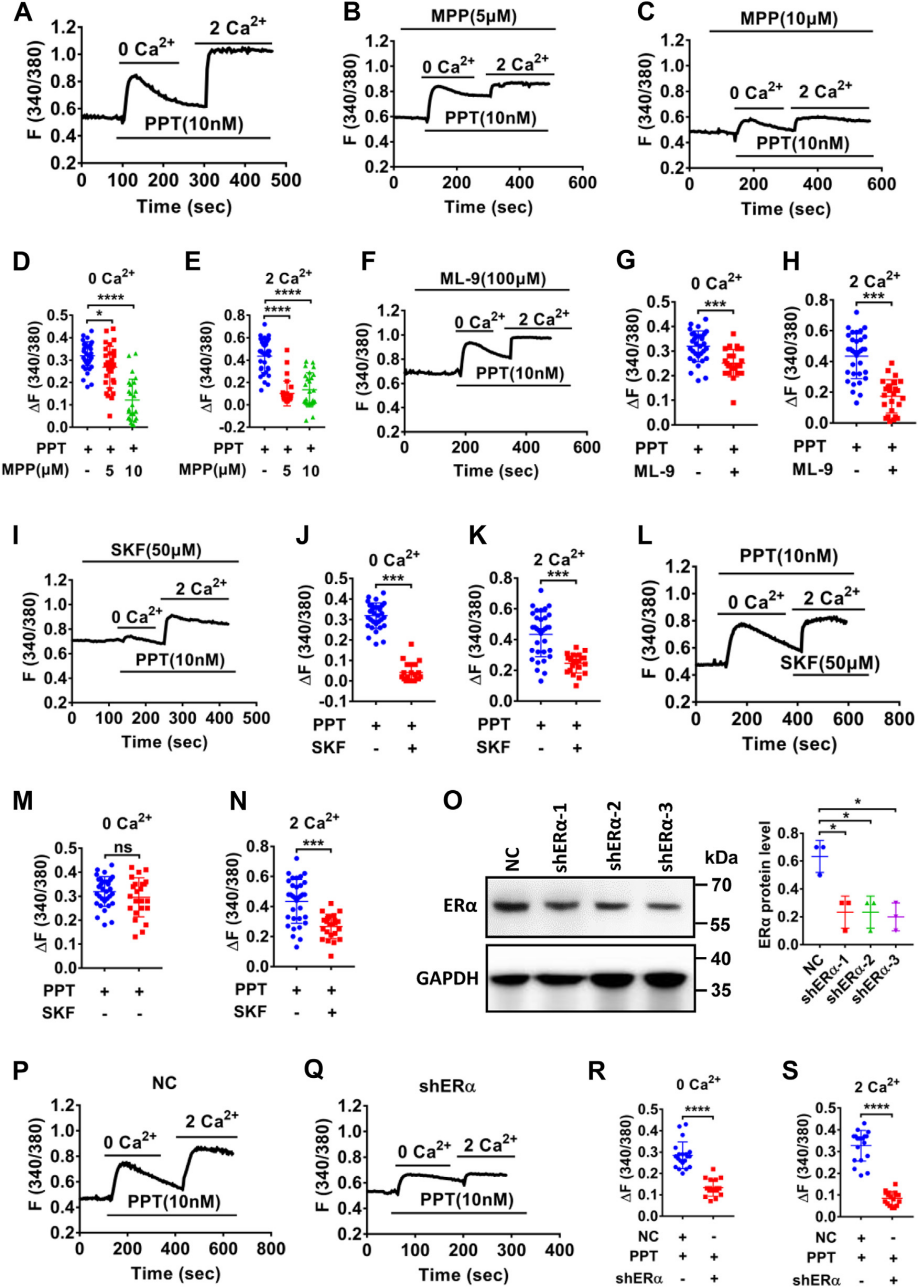
**ERα activation raised [Ca**^**2+**^**]**_**cyt**_***via* the SOCE in HCoEpiC**. *A*, summary tracings of [Ca^2+^]_cyt_ time course in response to PPT (10 nM, n = 33) in Ca^2+^-free solutions and in Ca^2+^-containing solutions. *B*, *C*, *F*, and *I*, summary tracings of [Ca^2+^]_cyt_ time course showing the inhibitory effect of MPP (5–10 μM, n = 30), ML-9 (100 μM, n = 25) and SKF96365 (50 μM, n = 21) on PPT-induced Ca^2+^ release and Ca^2+^ influx. *D* and *E*, summary data showing the peaks of PPT-induced [Ca^2+^]_cyt_ signaling with or without MPP as described in (*A*–*C*). *G* and *H*, summary data showing the peaks of PPT-induced [Ca^2+^]_cyt_ signaling with or without ML-9 as described in (*A* and *F*). *J* and *K*, summary data showing the peaks of PPT-induced [Ca^2+^]_cyt_ signaling with or without SKF96365 as described in (*A* and *I*). *L*, summary tracings of [Ca^2+^]_cyt_ time course showing the inhibitory effect of SKF96365 (50 μM, n = 23) on PPT-induced Ca^2+^ influx. *M* and *N*, summary data showing the peaks of PPT-induced [Ca^2+^]_cyt_ signaling as described in (*A* and *L*). *O*, Western blotting analysis of ERα proteins expression in NC and shERα (*left*) and the summary data of ERα protein expression (*right*) (n = 3). *P* and *Q*, summary tracings of [Ca^2+^]_cyt_ time course in response to PPT (10 nM) of NC (n = 20) and shERα-1 (n = 16) in Ca^2+^-free solutions and in Ca^2+^-containing solutions. *R* and *S*, summary data showing the peaks of PPT-increased [Ca^2+^]_cyt_ signaling with NC or shERα as described in (*P* and *Q*). ∗*p* < 0.05, ∗∗*p* < 0.01, ∗∗∗*p* < 0.001 and ∗∗∗∗*p* < 0.0001. ns, no significant differences. Data are presented as mean ± SD. The statistical significance of differences in the means of experimental groups was determined using Student's *t* test or one-way ANOVA followed by post hoc test for multiple pairwise comparisons. [Ca^2+^]_cyt_, free cytoplasmic Ca^2+^; ER, estrogen receptor; HCoEpiC, human colonic epithelial cell line; MPP, MPP dihydrochloride; PPT, propyl pyrazole triol; SOCE, store-operated Ca^2+^ entry.

We further elucidated the underlying mechanisms of ERα-induced Ca^2+^ signaling in HCoEpiC. Like SOCE inducer CPA, PPT (10 nM) significantly stimulated [Ca^2+^]_cyt_ in Ca^2+^-free solutions and then a sustained [Ca^2+^]_cyt_ increase in Ca^2+^-containing solutions (Fig. 7*A*). Further studies showed that either MPP dihydrochloride (MPP) at 5 to 10 μM (Fig. 7, *B*–*E*), a ERα selective inhibitor, or ML-9 (100 μM) (Fig. 7, *F*–*H*) and SKF96365 (50 μM) (Fig. 7, *I*–*K*), two different inhibitors of the SOCE, markedly eliminated PPT-induced Ca^2+^ release and Ca^2+^ influx, suggesting that ERα activation triggers [Ca^2+^]_cyt_ *via* the SOCE. Moreover, SKF96365 (50 μM) markedly reduced PPT-induced Ca^2+^ entry when it was added after the store depletion but before extracellular Ca^2+^ entry (Fig. 7, *L*–*N*). Finally, after shERα was applied to successfully knock down the protein expression of ERα in HCoEpiC (Fig. 7*O*), PPT-induced [Ca^2+^]_cyt_ was significantly attenuated by shERα-3 as well (Fig. 7, *P*–*S*), verifying ERα activation of the SOCE in colonic epithelial cells.

### ERα promoted Ca^2+^-dependent proliferation and migration of HCoEpiC

**Figure 8:**
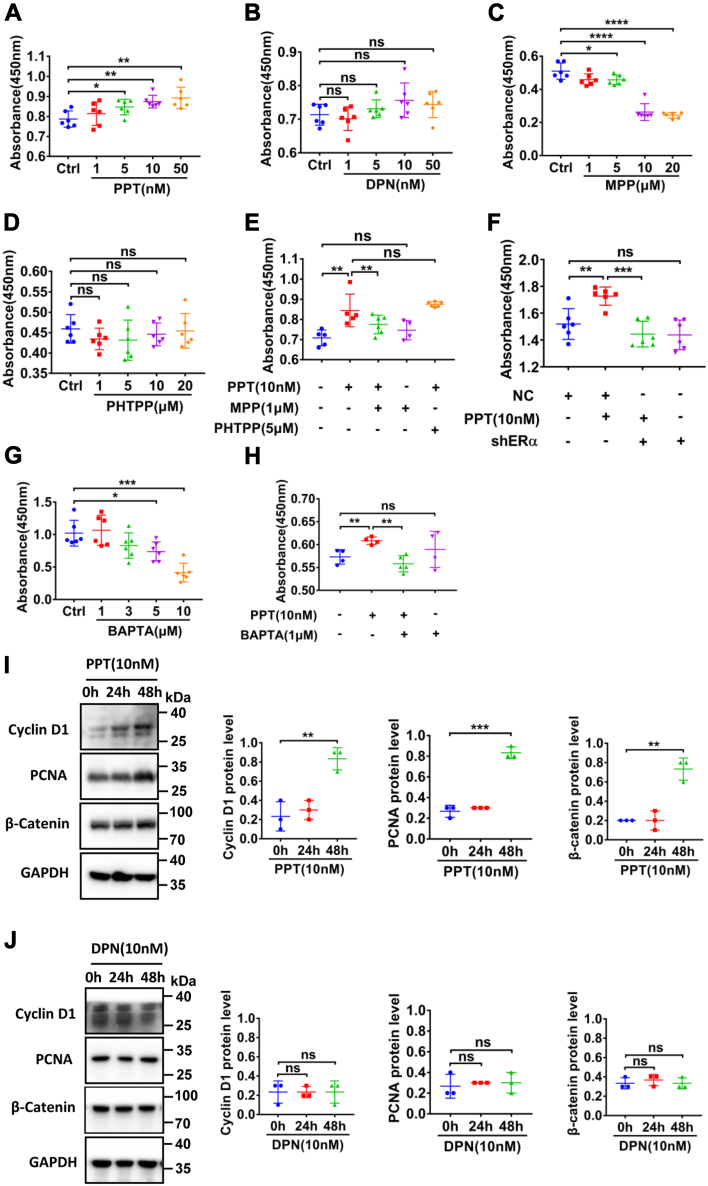
**ERα activation promotes Ca**^**2+**^**-dependent proliferation of HcoEpiC**. *A*, PPT (5–50 nM) dose-dependently enhanced cell proliferation. *B*, DPN (1–50 nM) did not affect cell proliferation. *C*, MPP (1–20 μM) dose-dependently inhibited cell proliferation. *D*, PHTPP did not affect cell proliferation. *E*, the inhibitory effect of MPP (1 μM) on PPT (10 nM)-induced cell proliferation. *F*, the inhibitory effect of shERα on PPT (10 nM)-induced cell proliferation. *G*, BAPTA-AM (1–10 μM) dose-dependently inhibited cell proliferation. *H*, the inhibitory effect of BAPTA-AM (1 μM) on PPT (10 nM)-induced cell proliferation. *I*, PPT (10 nM) at 48 h-enhanced cyclin D1, PCNA, and β-catenin protein expression. *J*, DPN (10 nM) did not affect protein expression of PCNA, cyclin D1, and β-catenin. ∗*p* < 0.05, ∗∗*p* < 0.01, ∗∗∗*p* < 0.001 and ∗∗∗∗*p* < 0.0001, ns, no significant differences. Data are presented as mean ± SD. The statistical significance of differences in the means of experimental groups was determined using Student's *t* test or one-way ANOVA followed by post hoc test for multiple pairwise comparisons. DPN, diarylpropionitrile; ER, estrogen receptor; HCoEpiC, human colonic epithelial cell line; MPP, MPP dihydrochloride; PCNA, proliferating cell nuclear antigen; PPT, propyl pyrazole triol.

Since proliferation and migration of epithelial cells are critical for the restitution of injured intestinal epithelium (33, 34), we examined the roles of ER subtypes in epithelial regeneration. PPT at 5 to 50 nM promoted proliferation of HCoEpiC (Fig. 8*A*), which was attenuated by ERα selective inhibitor MPP (1 μM, Fig. 8*E*) and shERα (Fig. 8*F*), but not by ERβ selective inhibitor PHTPP (5 μM) (Fig. 8*E*). However, DPN at 1 to 50 nM did not affect proliferation of HCoEpiC (Fig. 8*B*). Finally, [Ca^2+^]_cyt_ chelator BAPTA-AM (1 μM) also inhibited PPT-induced cell proliferation (Fig. 8*H*). MPP (1 μM), PHTPP (5 μM), and BAPTA-AM (1 μM) were chosen at these concentrations because they *per se* did not directly alter HCoEpiC proliferation (Fig. 8, *C*, *D*, and *G*).

Since cyclin D1, proliferating cell nuclear antigen (PCNA), and β-catenin play crucial roles in enterocyte proliferation (35, 36), we examined their protein expression following ER subtype stimulation. As shown in Fig. 8*I*, the pretreatment with PPT (10 nM) for 48 h enhanced the expression of cyclin D1, PCNA, and β-catenin. However, DPN (10 nM) did not affect their expression (Fig. 8*J*). Therefore, ERα promotes Ca^2+^-dependent proliferation of HCoEpiC.

**Figure 9:**
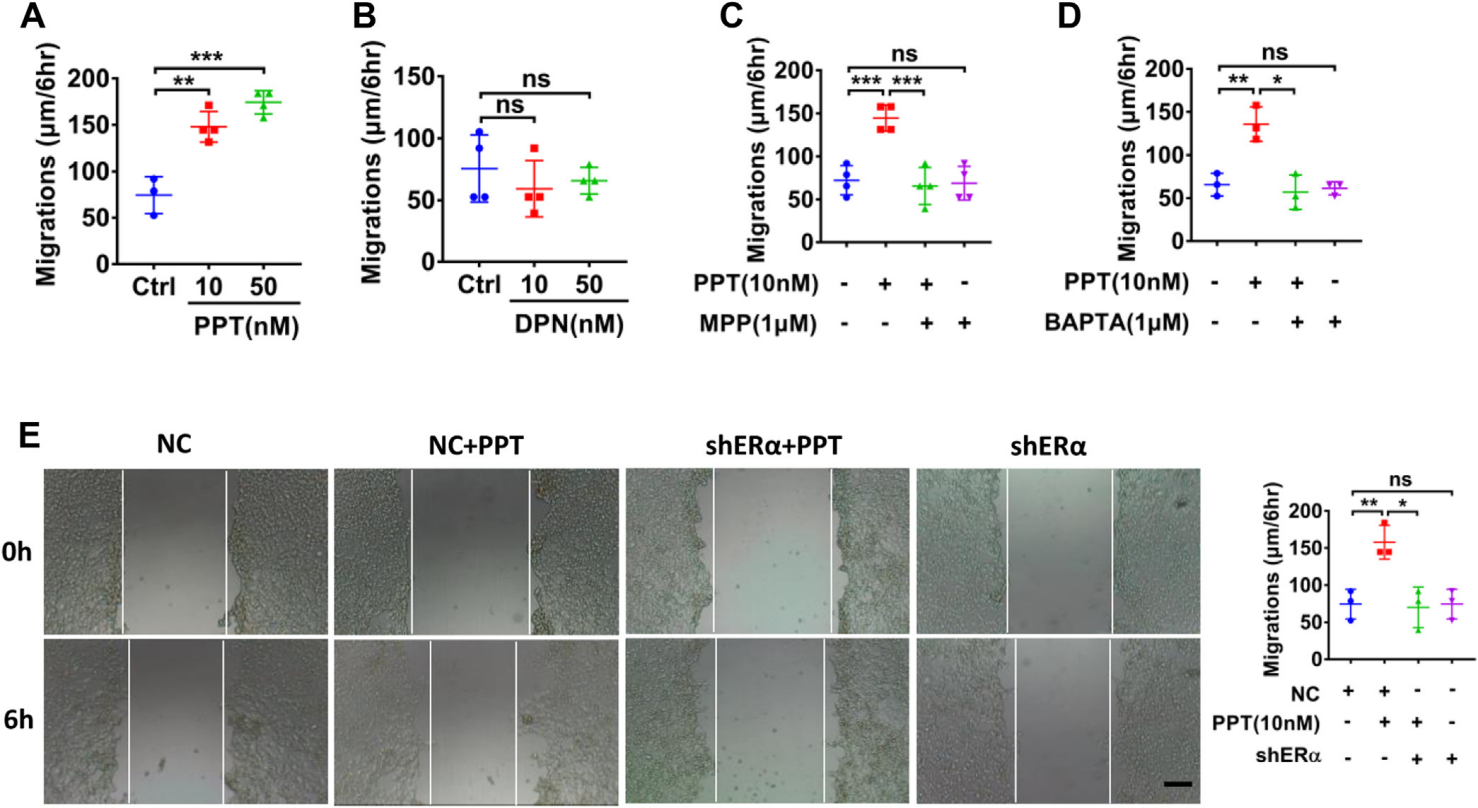
**ERα activation promotes HCoEpiC migration following injury**. *A*, PPT (10–50 nM) dose-dependently enhanced cell migration. *B*, DPN (10–50 nM) did not affect cell migration. *C*–*E*, the inhibitory effect of MPP (1 μM), BAPTA-AM (1 μM), and shERα on PPT (10 nM)-induced cell migration. The scale bar represents 200 μm for each image. ∗*p* < 0.05, ∗∗*p* < 0.01, and ∗∗∗*p* < 0.001. ns, no significant differences. Data are presented as mean ± SD. The statistical significance of differences in the means of experimental groups was determined using Student's *t* test or one-way ANOVA followed by post hoc test for multiple pairwise comparisons. DPN, diarylpropionitrile; ER, estrogen receptor; HCoEpiC, human colonic epithelial cell line; MPP, MPP dihydrochloride; PPT, propyl pyrazole triol.

Next, we performed cell scratch assays to examine the roles of ER subtypes in HCoEpiC migration. As shown in Fig. 9, *A* and *B*, PPT at 10 to 50 nM promoted cell migration, but DPN (10–50 nM) did not affect it. Moreover, ERα selective inhibitor MPP (1 μM) and BAPTA-AM (1 μM) and shERα abolished PPT-induced cell migration (Fig. 9, *C*–*E*). Therefore, ERα also promotes Ca^2+^-dependent migration of HCoEpiC.

### ER subtype expression in HCoEpiC and native mouse colonic epithelia

**Figure 10:**
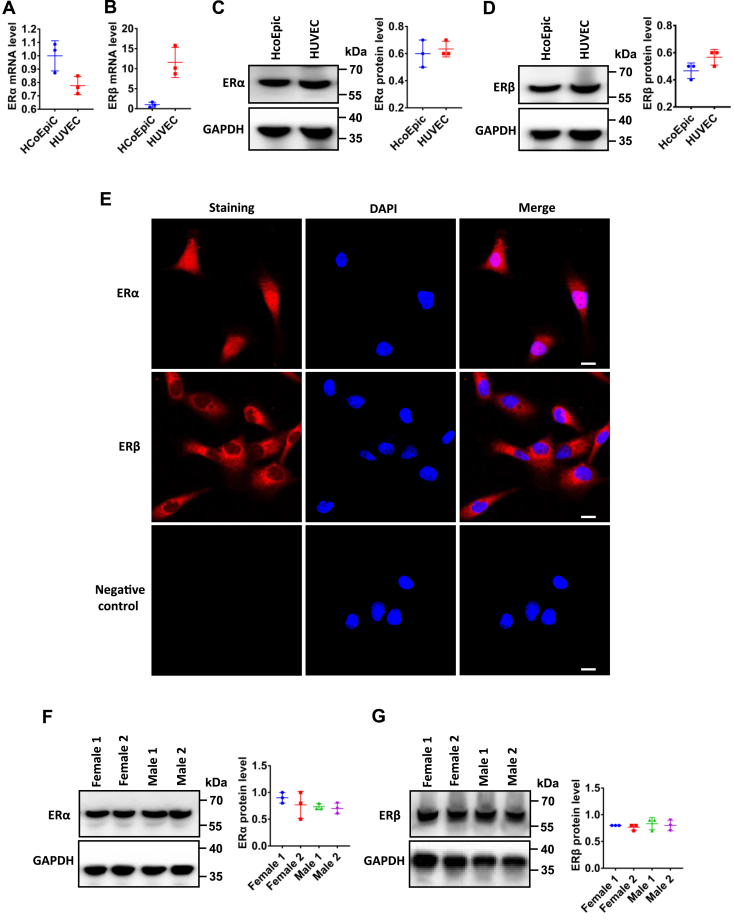
**The expression of ER subtypes in HCoEpiC and mouse colon**. *A* and *B*, RT-PCR showing mRNA expression of ERα and ERβ in HCoEpiC and HUVEC cells (n = 3). *C* and *D*, Western blots showing protein expression of ERα and ERβ in HCoEpiC and HUVEC cells and the summary data of ERα and ERβ protein expression (n = 3). *E*, the expression and localization of ERα and ERβ detected by immunofluorescence staining. Upper panel: ERα proteins staining (in *red*) and merge with nuclei stained with DAPI (in *blue*). Middle panel: ERβ proteins staining (in *red*) and merge with nuclei stained with DAPI. Lower panel: nuclei of the cells stained with DAPI without primary antibodies against ERα and ERβ as a negative control. The scale bar represents 20 μm for each image. *F* and *G*, representative Western blots and quantification of ERα and ERβ subtype protein expression in male and female mouse colon (n = 3). ER, estrogen receptor; HCoEpiC, human colonic epithelial cell line; HuVEC, human umbilical vein endothelial cell.

Since there is no information available in literature on ER subtype expression in HCoEpiC, we examined ER subtype expression in HCoEpiC and human umbilical vein endothelial cells (HUVECs) as a positive control (37, 38). As shown in Figure 10, *A* and *B*, both ERα and ERβ mRNA expression were detected in HCoEpiC. Western blots analysis further confirmed ERα and ERβ protein expression in HCoEpiC like in HUVEC (Fig. 10, *C* and *D*). Moreover, we also performed immunofluorescence to examine the expression and localization of ER subtypes in HCoEpiC. As shown in Figure 10*E*, ERα proteins were predominately expressed in the cytoplasm and nucleus, but ERβ were mostly expressed in the cytoplasm compared to the nucleus. However, the immunofluorescence staining was not observed without the primary antibodies against ERα and ERβ in the negative control, indicating specific staining on these proteins in HCoEpiC.

ERα and ERβ mRNA expression was previously detected in human and mouse colonic epithelia (38, 39, 40); however, the protein expression of these receptors in colonic epithelia is unknown. So, we performed Western blots analysis to examine protein expression of ER subtypes in mouse colonic epithelia. As shown in Figure 10, *F* and *G*, both proteins were expressed in native colonic epithelia in male and female mice, further supporting both ERα and ERβ expression in mice (38, 39).

## Discussion

**Figure 11:**
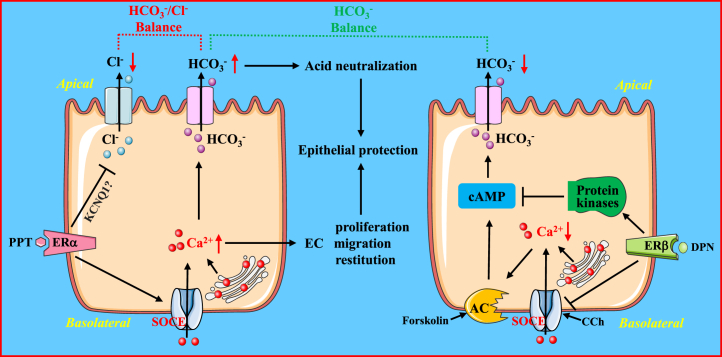
**Schematic depicting the proposed mechanisms of ER subtype–mediated colonic ion transport and epithelial restitution**. The *left*: ERα activation inhibits colonic Cl^−^ secretion but promotes colonic HCO_3_^−^ secretion and cell restitution to produce epithelial protection via the SOCE/Ca^2+^ signaling. The *right*: ERβ activation attenuates colonic HCO_3_^−^ secretion by blocking the SOCE/Ca^2+^ signaling to inhibit adenylyl cyclase (AC) and by activating protein kinases to inhibit cAMP signaling. ERα and ERβ subtypes orchestrate the homeostasis of colonic epithelial ion transports. ER, estrogen receptor; EC, epithelial cell; SOCE, store-operated Ca^2+^ entry; PPT, propyl pyrazole triol; DPN, diarylpropionitrile; KCNQ1, a potassium channel.

Although sex-based differences in E_2_ regulation of intestinal Cl^−^ and HCO_3_^−^ secretion have previously been described (9, 10, 14, 15, 16), it has been unknown what specific ER subtypes may be responsible for intestinal ion transport and epithelial restitution. In the present study, for the first time, we demonstrate ER subtypes in E_2_-mediated colonic ion transports and epithelial restitution in distinct ways (please see Fig. 11). Here we show that (1) both ERα and ERβ subtypes are expressed in HCoEpiC and mouse colonic epithelia, (2) ERα attenuates E_2_-mediated colonic Cl^−^ secretion but promotes E_2_-mediated HCO_3_^−^ secretion *via* SOCE/Ca^2+^ signaling, (3) ERβ attenuates E_2_-mediated HCO_3_^−^ secretion by inhibiting SOCE/Ca^2+^ signaling and by inhibiting cAMP signaling *via* activation of TK, PKC, and PI3K, (4) Erα but not Erβ promotes E_2_-mediated epithelial cell restitution *via* the SOCE, and (5) ERα enhances the expression of cyclin D1, PCNA, and β-catenin in HCoEpiC.

We previously reported that E_2_ stimulated murine duodenal HCO_3_^−^ secretion (9) and revealed gender differences in human duodenal HCO_3_^−^ secretion (10), strongly supporting a protective role of E_2_ against mucosal acid exposure in the duodenum of females. Although our studies have provided a potential explanation for the lower prevalence of duodenal ulcer in women (9, 10), so far it is unclear whether E_2_ plays these roles *via* different ER subtypes (ERα and ERβ) to exert various biological actions through genomic and nongenomic pathways (41, 42). Genomic pathway involves nucleus/transcription factors to modulate specific gene expression, but nongenomic pathway induces immediate responses through multiple signaling pathways, such as Ca^2+^, cAMP, TK, PKC, and PI3K pathways (42). Although genomic action of E_2_ has been well studied, its nongenomic action on GI function is less understood. In the present study, we found divergent roles for ERα and ERβ subtypes in regulating colonic ion transport and HCO_3_^−^ secretion. ERα activation promoted immediate E_2_-mediated HCO_3_^−^ secretion but did not alter CCh- and forskolin-stimulated HCO_3_^−^ secretion, suggesting a direct activation of ERα *via* nongenomic action. However, although ERβ activation alone did not affect colonic HCO_3_^−^ secretion, it attenuated CCh- and forskolin-stimulated colonic HCO_3_^−^ secretion likely *via* protein kinases (22, 25).

Most well-known secretagogues, such as forskolin and CCh usually stimulate both epithelial HCO_3_^−^ and Cl^−^ secretion in parallel (43, 44), but in the case of E_2_, it appears that Cl^-^ and HCO_3_^−^ secretion may be regulated in distinct ways. Although E_2_ inhibited colonic Cl^−^ secretion through KCNQ1 channels (14, 15, 28), we showed here E_2_ rapid stimulation of HCO_3_^−^ secretion *via* nongenomic action through distinct effects of ERα and ERβ subtypes on intracellular signaling responsible for colonic Cl^-^ and/or HCO_3_^-^ secretion. Since E_2_ could promote duodenal HCO_3_^−^ secretion *via* Ca^2+^ signaling (9), it is reasonable to infer that ER may promote Ca^2+^-dependent HCO_3_^−^ secretion. Ca^2+^ enters nonexcitable epithelial cells mainly through the SOCE, and its critical role has been descried in intestinal epithelial cells (45, 46, 47, 48). In the present study, we found that ERα promotes but ERβ inhibits SOCE/Ca^2+^ signaling, suggesting divergent roles of ER subtypes in regulating SOCE/Ca^2+^ signaling.

Interestingly, ERβ activation did not affect basal colonic HCO_3_^−^ secretion but markedly attenuated CCh- and forskolin-stimulated HCO_3_^−^ secretion. Since it is well-known that CCh and forskolin stimulated HCO_3_^−^ secretion *via* Ca^2+^ and cAMP signaling, respectively, we further elucidated the underlying mechanisms of ERβ-inhibited colonic HCO_3_^−^ secretion *via* Ca^2+^ and cAMP pathways. We not only found that ERβ inhibited SOCE mechanism (49) but also revealed that inhibition of protein kinases reverses ERβ-inhibited colonic HCO_3_^−^ secretion *via* cAMP pathway, suggesting that ERβ-activated protein kinases are involved in this process. In contrast, inhibition of protein kinases did not reverse ERα-inhibited HCO_3_^−^ secretion *via* Ca^2+^-pathway, further supporting the notion that ERβ inhibits colonic HCO_3_^−^ secretion by suppressing Ca^2+^ signaling *via* SOCE and cAMP signaling *via* protein kinases.

We previously showed that activation of the Ca^2+^-sensing receptor in the duodenum resulted in [Ca^2+^]_cyt_ increase but cAMP decrease (19); the end result being increased duodenal HCO_3_^−^ secretion without simultaneously altering *I*_*sc*_. Therefore, the data from Ca^2+^-sensing receptor and ER further support our notion that epithelial HCO_3_^−^ and Cl^−^ secretion could be triggered differentially by Ca^2+^ and cAMP signaling (9, 19). Determining how to specifically modulate Cl^−^ *versus* HCO_3_^−^ secretion is important as one develops drugs to improve acid–base balance and epithelial repair in GI diseases (*e.g.*, duodenal ulcer disease, cystic fibrosis, ulcerative colitis [UC]) without triggering excessive Cl^−^ secretion that might induce the unwanted diarrheal side effects.

While epithelial restitution plays a critical role in the healing process of intestinal mucosa (17), little is known about the role of ER in colonic mucosal healing. Diseases like UC, a global intestinal autoimmune disease with no available cure, require an ongoing process of healing from injury. The foundational target of UC therapies is to suppress the immune system, thereby leading to less autoreactivity. However, large amounts and long-term immunosuppressive therapy increases the risk for infections and cancers (50). Therapies that may promote mucosal healing without immunosuppression are enticing as stand-alone or adjunctive therapies. In addition to modulating epithelial ion transport, ERα, but not ERβ, activation promotes Ca^2+^-dependent cell proliferation and migration, leading to colonic epithelial restitution; a process that may be beneficial to colonic disease like UC. Moreover, we found that activation of ERα but not ERβ increases protein levels of cyclin D1, PCNA, and β-catenin, indicating increased proliferation and migration.

In conclusion, we demonstrate for the first time that E_2_ modulates colonic epithelial ion transports and epithelial restitution *via* ER subtype-dependent mechanisms. Since ERα and ERβ subtypes usually play opposite roles in regulating colonic epithelial function, E_2_ may coordinate different ER subtypes to orchestrate functional homeostasis of colonic epithelial cells. Disruption of ERα- and ERβ-coordinated gut homeostasis may have underpinnings behind sex-based differences in GI disease, especially those involving epithelial injury and repair. While this requires further study, we have provided new insights into the cellular mechanisms of E_2_-mediated colonic epithelial ion transports and epithelial repair *via* different ER subtypes.

## Experimental procedures

### Cell culture

Human colonic epithelial cells (HCoEpiC) (Cat #2950) and HUVEC (Cat #8000) were purchased from ScienCell Research Laboratory. Cells were cultured in DMEM-HIGH GLUCOSE or RPMI-1640 (HyClone) supplemented with 10% fetal bovine serum (HyClone) in a 37 °C humidified atmosphere containing 5% CO_2_.

### Animal studies and ethics

All animal studies were approved by the Ethics Committee of the Qingdao University Medical College. All animal care and experimental procedures complied with the “Guide for the Care and Use of Laboratory Animals” published by the National Institutes of Health. Animal studies are reported in compliance with the ARRIVE guidelines (51). The C57BL/6 mice (6–8 weeks old; 18–22 g) were purchased from HFK Bioscience Co., Ltd. Animals were assigned randomly to different experimental groups. Randomization and single-blinding were used for the measurement.

### Measurement of intestinal *I*_*sc*_ and HCO_3_^−^ secretion in Ussing chamber experiments

Ussing chamber experiments were performed as previously described (52, 53). C57BL/6J mice were anesthetized by halothane, and the abdomen was opened with a midline incision. Duodenal and colonic tissues were removed, stripped of seromuscular layers, divided, and mounted in Ussing chambers (aperture area, 0.1 cm^2^). The experiments were performed under continuous short-circuited conditions (Voltage-Current Clamp, VCC MC6; Physiologic Instruments), and luminal pH was maintained at 7.0 by the continuous infusion of 5 mm HCl under the automatic control of a pH-stat system (ETS 822; Radiometer America). The volume of the titrant infused per unit time was used to quantitate HCO_3_^−^ secretion. Measurements were recorded at 5-min intervals, and mean values for consecutive 5- or 10-min periods were averaged. The rate of luminal HCO_3_^−^ secretion is expressed as micromoles per square centimeter per hour. The transepithelial *I*_*scs*_ were measured *via* an automatic voltage clamp, in which μA was used for the original recordings, but μA·cm^-2^ was used for summary data. After a 30 min basal period, inhibitor or control vehicle was added for another 30 min, followed by addition of stimulus to both sides of the tissue. Electrophysiological parameters and HCO_3_^−^ secretion were then recorded for 60 min. The mucosal solution contained the following (mM): 115 NaCl, 25 sodium-D-gluconate, 5.2 potassium-D-gluconate, 1.2 CaCl_2_, 1.2 MgCl_2_, and 10 mannitol. The serosal solution contained the following (mM): 115 NaCl, 25 NaHCO_3_, 2.2 K_2_HPO_4_, 1.2 CaCl_2_, 1.2 MgCl_2_, 0.8 KH_2_PO_4_, 10 glucose, and 0.01 indomethacin. The osmolalities for both solutions were ∼300 mosmol·kg^-1^ of H_2_O.

The maximal volumes of concentrated stock solutions of all compounds added to 3 ml Ussing chamber solutions were less than 30 μl. All added compounds did not alter pH values in chamber solutions, which is consistent with other report (25). The concentrations of all pharmacological inhibitors used in the present study were based on their IC_50_ and the data from others’ reports.

### Measurement of [Ca^2+^]_cyt_ by single-cell imaging

[Ca^2+^]_cyt_ imaging experiments were performed as previously described (52). Briefly, cells were grown on glass coverslips for 24 h and incubated with 5 μM fura-2/AM (Invitrogen) for 1 h in physiological salt solution (PSS) at 37 °C humidified atmosphere containing 5% CO_2_ in the dark and then washed with PSS for 20 min. Then, cells on coverslips were mounted in a standard perfusion chamber on the stage of an inverted fluorescence microscope (Leica). Fluorescence signals were imaged using an intensified CCD camera (ICCD200) attached to an inverted fluorescence microscope (Leica) and recorded with MetaFluor software (Universal Imaging Corporation). Images were acquired every 3 s. The dual wavelength excitation method for the measurement of fura-2 fluorescence was used. The excitation wavelengths were 340 and 380 nm, and the emitted fluorescence was collected at 510 nm. [Ca^2+^]_cyt_ was presented as fluorescence ratios (F340/F380) after background subtraction. The PSS contained the following: 140 mM Na^+^, 5 mM K^+^, 2 mM Ca^2+^, 147 mM Cl^−^, 10 mM Hepes and 10 mM glucose (pH 7.4). The 0 Ca^2+^ solution (0 Ca^2+^) contained the following: 140 mM Na^+^, 5 mM K^+^, 145 mM Cl^−^, 0.5 mM EGTA, 10 mM Hepes, and 10 mM glucose (pH 7.4). The osmolality for the solution was ∼300 mosmol^.^kg^−1^ of H_2_O.

### Western blotting

Western blotting was performed as previously described (52, 54). Briefly, the cells and tissues were harvested and lysed using RIPA lysis buffer. The protein sample were separated using 4 to 20% SDS-PAGE and transferred to polyvinylidene difluoride membrane. Following blocking with 5% nonfat milk for 2 h at room temperature, the membranes were incubated with the following primary antibodies at 4 °C overnight: anti-ERα (1:1000) (Cat. No. ab32063, Abcam), anti-ERβ (1:1000) (Cat. No. PA1-311, ThermoFisher) antibody, and anti-GAPDH (1:10,000) (Cat. No. 60004-1-Ig, Proteintech), anti-β-catenin (1:1000) (Cat. No. 8480, Cell Signaling Technology), anti-Cyclin D1 (1:1000) (Cat. No. 2978, Cell Signaling Technology), anti-PCNA (1:1000) (Cat. No. ab29, Abcam). Then, the membranes were washed with Tris-buffered saline with 0.1% Tween 20 detergent three times and incubated with corresponding secondary antibodies for 2 h at room temperature. The signals were visualized using enhanced chemiluminescence (Millipore) in an ImageQuant LAS 400 digital biomolecular imaging system. Each experiment was repeated three times. The gray value of the bands was measured by ImageJ software for statistics.

### Quantitative real-time PCR

Quantitative real-time PCR was performed as previously described (54, 55). Briefly, Total RNA was extracted by RNAiso Plus reagent (Cat. No. 9109, Takara). cDNA was synthesized using PrimeScript RT-polymerase (Cat. No. R050A, Takara). Next, qPCR was performed using a SteponePlus device (Art. No. 272008342, Life Technologies) with a SYBR Premix Ex TaqTM II kit (Cat. No. RR820A, Takara). All samples were run in triplicate, and GAPDH was used as an internal control. Primers were as follows:

ERα: 5′- GGGAAGTATGGCTATGGAATCTG-3′ (Forward),

ERα: 5′- TGGCTGGACACATATAGTCGTT-3′ (Reverse).

ERβ: 5′-AGCACGGCTCCATATACATACC-3′ (Forward),

ERβ: 5′- TGGACCACTAAAGGAGAAAGGT-3′ (Reverse).

GAPDH: 5′- ACAACTTTGGTATCGTGGAAGG-3′ (Forward),

GAPDH: 5′- GCCATCACGCCACAGTTTC-3′ (Reverse).

### Immunofluorescence staining

After fixed with 4% paraformaldehyde, permeabilized with 0.25% Triton X-100, and blocked with bovine serum albumin, the HCoEpiC were incubated with anti-ERα (1:100) (Cat. No. ab32063, Abcam) or anti-ERβ (1:200) (Cat. No. PA1-311, ThermoFisher) antibody overnight at 4 °C. Then, the cells were incubated with Cy3 labeled anti-rabbit secondary antibody (Cat. No. A0516, Beyotime Biotechnology). Finally, nuclei were stained with DAPI for 5 min, and images were captured using confocal microscope.

### Cell proliferation and scratch assays

Cell proliferation assay was performed as previously described (56). Briefly, cells were plated in 96-well plates. After 24 h, medium was replaced with medium containing different drugs. CCK-8 reagent (Cat. No. C0038, Beyotime Biotechnology) was added to each well at 0.5 to 2 h before the endpoint of incubation. A microplate reader (Thermo Fisher Scientific) was used to quantify viable cells by measuring the absorbance at 450 nm. Cell scratch assay was performed as previously described (57). After scratching, cell monolayers were gently washed to remove detached cells and replenished with serum free medium (with or without drugs) to inhibit cell proliferation. Images were obtained at 0 and 6 h postscratch. Experiments were repeated at least three times.

### Infection of lentiviruses

Lentiviruses were purchased from Sangon Biotech Co, Ltd. The sequences for ERα shRNA and NC were as follows: shRNA-1 (5′-TTGTGTGCCTCAAATCTATTA-3′), shRNA-2 (5′-AGGCCAAATTCAGATAATCGA-3′), shRNA-3 (5′- CAGGTCCACCTTCTAGAATGT-3′), shNC (5′- TTCTCCGAACGTGTCACGT -3′). HCoEpiC were infected with lentiviruses according to the protocol of the manufacturer.

### Drugs

PPT, DPN, MPP, PHTPP, E_2_, forskolin, genistein, rottlerin, wortmannin, GSK-7975A, ML-9, SKF-96365, CPA, and BAPTA-AM were purchased from MedChemExpress and dissolved in DMSO. CCh and indomethacin were purchased from Sigma-Aldrich. CCh was dissolved in ultrapure water. Indomethacin was dissolved in anhydrous alcohol. All salts were supplied by Sangon Biotech and dissolved in ultrapure water.

### Data and statistical analysis

GraphPad Prism 7.0 (RRID: SCR_002798, USA) software was used for analysis and graph generation. All results shown are means ± SD. All experiments were repeated at least three times. The number of biological repeats (n) in the figures is the number of individual tissues or cells obtained from at least three mice or three independent experiments. The statistical significance of differences in the means of experimental groups was determined using Student's *t* test or one-way ANOVA followed by post hoc test for multiple pairwise comparisons. Significant differences (∗*p* < 0.05) are expressed in the figures and figure legends.

## Data availability

The data supporting the findings of this study are available within the article.

## Conflicts of interest

The authors declare no conflicts of interest with the content of this article.
